# Enhancing resiliency and optimizing readiness in military personnel through psychological flexibility training: design and methodology of a randomized controlled trial

**DOI:** 10.3389/fpsyt.2023.1299532

**Published:** 2024-01-05

**Authors:** Alan L. Peterson, Brian A. Moore, Wyatt R. Evans, Stacey Young-McCaughan, Abby E. Blankenship, Casey L. Straud, Christopher S. McLean, Tashina L. Miller, Eric C. Meyer, for the STRONG STAR Consortium

**Affiliations:** ^1^Department of Psychiatry and Behavioral Sciences, University of Texas Health Science Center at San Antonio, San Antonio, TX, United States; ^2^Research and Development Service, South Texas Veterans Health Care System, San Antonio, TX, United States; ^3^Department of Psychology, University of Texas at San Antonio, San Antonio, TX, United States; ^4^Department of Psychological Science, Kennesaw State University, Kennesaw, GA, United States; ^5^VA North Texas Health Care System, Dallas, TX, United States; ^6^Department of Psychiatry, University of Texas Southwestern Medical Center, Dallas, TX, United States; ^7^3rd Security Force Assistance Brigade, Fort Cavazos, TX, United States; ^8^Department of Counseling and Behavioral Health, University of Pittsburgh, Pittsburgh, PA, United States

**Keywords:** resilience, psychological flexibility, acceptance and commitment therapy, military personnel, military optimization, readiness

## Abstract

**Background:**

Enhancing resiliency and optimizing readiness in military personnel is a high priority for the U.S. Department of Defense. Most military resiliency-enhancement programs are evidence-informed interventions. However, few randomized studies have demonstrated efficacy of any intervention or training program to enhance resiliency and prevent the development of psychological health symptoms in military personnel when exposed to operational stressors. This manuscript provides an overview of the theoretical foundation, research design, and research methods of a preventive intervention trial designed to evaluate the efficacy of a training program to enhance resiliency and prevent psychological health symptoms in military personnel. The resiliency training intervention is based on Acceptance and Commitment Therapy (ACT), an evidence-based intervention with broad empirical support for improving functioning in those living with psychological and medical conditions.

**Method/design:**

This study will evaluate the efficacy of a two-day training program based on ACT for fostering psychological flexibility, the central target in ACT, for enhancing resiliency, and for preventing the development of psychological health symptoms. The research participants will be a non-clinical population of active duty military personnel (*N* = 600). The ACT-based training program (*n* = 300) will be compared to a military resiliency training as usual, known as Master Resilience Training (*n* = 300). Assessment measures will be administered at the baseline assessment, after training, prior to a military deployment, and after returning from a deployment. Qualitative interviews will be conducted to provide feedback on the training program.

**Clinical Trial Registration:** NCT05094115.

## 1 Introduction

Maintaining health, optimizing performance, and enhancing resiliency to occupational stressors in military personnel who are frequently exposed to austere environments and trauma is a significant challenge. The Department of Defense has invested significant resources to develop and evaluate programs to enhance resiliency, optimize operational readiness, and prevent deployment-related psychological health casualties in military personnel (1). A limited amount of previous literature has shown positive effects of self-reflection on the subsequent development of mental health symptoms in Australian military cadets (2, 3) and on posttraumatic stress symptoms following a debriefing intervention among recently deployed U.S. soldiers (4). These studies highlight the importance of using a valid control group, as well as the importance of implementing interventions to prevent or mitigate the development of psychological health symptoms in military personnel when exposed to occupational or operational stressors. Most military resiliency-enhancement programs, such as the U.S. Army’s Comprehensive Soldier Fitness Program, are evidence-informed interventions that are implemented as population health programs (5, 6). Indeed, the effectiveness of the Comprehensive Soldier Fitness Program, which is the Army’s current resiliency-enhancement program of record, has not been supported based on the results of controlled trials (7).

There are several reasons for the lack of rigorous (i.e., prospective, randomized controlled) resiliency-enhancement trials in U.S. military populations. First, scientifically evaluating the potential efficacy of a resiliency-enhancement training program requires a prospective study in which military personnel are randomized to different arms of a controlled intervention trial, which can be difficult in the contemporary operating environment. Second, various groups of military personnel might be ideal populations to study, but with each presenting with unique challenges. For example, one population might be new military recruits who are enrolled, randomized, trained, and evaluated as they complete basic military training, a demanding and highly stressful environment (8). Another population might be military special forces trainees who are about to start a training program, such as the U.S. Navy’s Basic Underwater Demolition/Sea, Air, and Land Teams (SEALs) course, which has been found to have a washout or dropout rate of about 80% (9, 10). Such intense training programs would provide a proxy for a high-stress environment that might be encountered during a deployment. However, most military trainees are considered protected populations, and their training programs are part of established training curricula that require high levels of military coordination and approval to modify for the purpose of conducting research. A final group who might be ideal to study are individuals who are scheduled to deploy to a high-risk/high-threat environment. However, designing and obtaining research regulatory review approval for a prospective study such as this would be extremely difficult, especially with the discontinuation of U.S. military combat deployments in support of Operation Iraqi Freedom in 2010 and Operation Enduring Freedom in 2014 (11). A rigorous schedule of deployment preparation activities makes adding something such as supplemental resilience training a challenge.

The current manuscript is a description of the methods for a research project funded through the U.S. Department of Defense’s Military Operational Medicine Research Program’s Resilience and Readiness Optimization/Enhancement (R2OE) Translational Research Award (W81XWH1910628; PI: Alan Peterson). The aim of the project is to conduct a randomized controlled trial evaluating the efficacy of psychological flexibility training based on Acceptance and Commitment Training to enhance resiliency in military personnel.

### 1.1 Acceptance and commitment therapy and acceptance and commitment training

Acceptance and Commitment Therapy (ACT; pronounced as one word) is a contemporary, evidence-based, cognitive-behavioral approach that was originally developed to improve psychological health and functioning in those experiencing psychological health challenges (12, 13). It is also a promising psychological approach for resilience enhancement in adults (14, 15). We recently described the potential of this model for fostering resilience enhancement in military personnel (16). The present grant was funded to adapt and evaluate a military-relevant psychological flexibility training intervention based on ACT to enhance resilience and optimize readiness.

There are over 1,000 published randomized controlled trials (RCTs) of ACT for improving a broad range of biopsychosocial outcomes. These RCTs span applications of ACT for depression, anxiety disorders, substance use disorders, psychosis, tobacco use, eating disorders, general well-being, and a range of medical challenges (17–24). ACT has been found to optimize functional outcomes in diverse domains including improving workplace performance under stressful conditions, completing physical exercise regimens, improving parenting skills, and adhering to dietary restrictions (15). Moreover, ACT has been found to be efficacious when delivered by individuals from different professional disciplines and in a broad range of formats including individual and group psychotherapy, brief training workshops, and online delivery. This underscores the robustness of the model and the potential for scalability (25–28). When used in a training context, ACT may be referred to as Acceptance and Commitment Training.

In ACT, the broad goal is to help individuals identify and act consistently with their personal values and goals and to align their actions with those values even if they are experiencing psychological or physical discomfort (9, 10). According to the ACT model, suffering and impairment are primarily the consequence of psychological inflexibility (i.e., inability to persist in or change behavior according to situational or contextual factors and personally chosen values due to problematic, inflexible reactions to negatively evaluated internal experiences). Psychological inflexibility may be particularly detrimental when an individual is confronted with stress or adversity (12, 13). ACT also incorporates mindfulness as a method for increasing awareness of and for promoting a nonjudgmental stance toward unwanted internal experiences, as these are key elements of fostering psychological flexibility (12). Interventions targeting increased psychological flexibility—ACT being the foremost in this field—are recommended not only for facilitating recovery after stressors but also for enhancing resilience (14, 16, 29, 30). The psychological flexibility model aligns well with the flexibility sequence model, arguably the most thoroughly researched and well supported model of the resilience process across numerous populations (31).

## 2 Materials and methods

### 2.1 Institutional review board

This project has been reviewed and approved by the Institutional Review Board (IRB) at the University of Texas Health Science Center at San Antonio (UTHSCSA; 20210486HU) as the IRB of record. The University of Pittsburgh deferred its review to UTHSCSA as part of a single IRB authorization agreement.

### 2.2 Design

The present RCT (*N* = 600) was designed to evaluate the efficacy of a novel, military-relevant, ACT-based resilience training that we refer to as Psychological Flexibility Training and that we developed for this project. The primary goal of this intervention is to enhance resiliency and optimize readiness in military personnel. For the proposed study, it is hypothesized that the ACT principles will also improve the psychological health outcomes by reducing symptoms of anxiety, depression, and posttraumatic stress disorder (PTSD) in military operational settings, as suggested by the Cochrane Database of Systematic Reviews on Psychological Interventions for Resilience Enhancement in Adults (14). We have two primary research hypotheses. First, we hypothesize that military personnel who receive psychological flexibility training will demonstrate a statistically significant increase in psychological resiliency compared to military personnel receiving training as usual over the course of the study. Second, we hypothesize that military personnel who receive Psychological Flexibility Training will demonstrate a statistically significant increase in psychological resiliency after completing a military operational deployment compared to a group of military personnel receiving military resilience training as usual. Military training as usual for the Army is Master Resilience Training, which is the resilience enhancement component of the broader Comprehensive Soldier Fitness Initiative (32). Of note, all participants receive Master Resilience Training as part of their training as usual. Thus, the current study is testing the incremental value of adding Psychological Flexibility Training.

#### 2.2.1 Randomization

Recruited personnel will be randomized as a group to Resilience Enhancement through Acceptance and Commitment Training (REACT) or regular programming. Group randomization will be completed prior to recruitment or consent. Groups will be randomized by a randomization list generated by the research team’s Biostatistics Core. The randomization list will use random blocks of 4, 6, and 8 to ensure roughly equal numbers of groups in each condition as the study progresses and to reduce the anticipation of group assignment and thereby the potential for bias.

### 2.3 Participants

#### 2.3.1 Source of population

Active duty U.S. Army soldiers assigned to Fort Cavazos (formerly Fort Hood), Texas, will be eligible to participate in the R2OE study. One unit that has already agreed to participate representing a military unit regularly scheduled to deploy to high-risk/high-threat environments is the 3rd Security Force Assistance Brigade (SFAB). SFABs are specialized Army units formed to train, advise, assist, enable, and accompany operations with allied and partner nations. They were created with the intent to reduce the burden of such operations on conventionally organized Brigade Combat Teams (BCTs), allowing BCTs to focus on fighting near-peer threats. Designed on the model of a standard infantry BCT, SFABs are composed of approximately 800 experienced senior personnel, primarily commissioned, warrant, and non-commissioned officers. They are selected from regular Army units and given additional training at the Military Advisor Training Academy at Fort Moore, Georgia. We will begin recruitment efforts with the 3rd SFAB. Should recruitment from SFABs prove insufficient, we will recruit from additional units stationed at Fort Cavazos, such as Brigade Combat Teams and other units that most closely resemble the SFABs in terms of military service characteristics.

### 2.4 Recruitment & randomization

Soldiers are recruited through briefings at various unit meetings as well as at the unit’s Newcomers’ Briefing held monthly. Any soldier, 18 years or older, is eligible for inclusion. There are no exclusion criteria. Participants are equally randomized between the two groups stratifying for rank to ensure equal numbers of junior non-commissioned officers (NCOs; grades E-4 to E-6), senior NCOs (grades E-7 to E-9), and officers (warrant and commissioned) are in each group. In accordance with Department of Defense (DoD) Instruction 3216.02, participants can be compensated if study participation does not adversely impact their ability to perform their assigned duties. Five assessments are scheduled. Each time an assessment is completed, $25 can be compensated, for a total of $125.

### 2.5 Consent

An authorized and trained member of the research team engages potential participants in an interactive explanation of the study guided by the informed consent document. Individual informed consent is obtained following recruitment briefings or at another time when a member of the research team can meet with interested individuals. Soldiers who are not interested in participating in the study continue with unit training as usual.

### 2.6 Measures

Ideally, resiliency enhancement training for military personnel demonstrates not only improvements in self-reported resiliency and other related constructs but also demonstrates positive adaptation in the face of significant stress or adversity as a result of the training (33). Related to this, the present study examines a range of resilience-related constructs (i.e., dispositional recovery, stress response, and grit) and correlates (dysfunctional cognitions, depression, and anxiety). Given our theoretical approach, we anticipate that resilience-related outcomes would be associated with increased psychological flexibility (16). The schedule of assessments is outlined in Table 1. A brief description of each measure is provided below. A more detailed description of each measure including information on validity and reliability of each measure is included in Supplement 1. Administered assessments address the constructs of psychological resilience, trait resilience, psychological flexibility, self-efficacy, mindset, life satisfaction, psychological health, unit cohesion, family functioning, and evaluation and feedback about the training (see Table 1).

**Table 1:** Schedule of assessments involved in the Resilience and Readiness Optimization/Enhancement (R2OE) study.

Construct	Assessment	Number of items	Baseline	Every 4 months
Psychological resilience	Connor-Davidson Resilience Scale (CD-RISC)	10	X	X
	Dispositional Recovery and Dysfunction Inventory (DRDI)	14	X	X
	Response to Stressful Experiences Scale (RSES)	22	X	X
Trait resilience	Short Grit Scale (Grit-S)	8	X	X
Psychological flexibility	Acceptance and Action Questionnaire-II (AAQ-2)	7	X	X
	Multidimensional Psychological Flexibility Inventory	60	X	X
Self-efficacy	General Self-Efficacy Questionnaire–6 (GSEQ-6)	6	X	X
Mindset	Implicit Theories of Intelligence Scale (ITIS)	6	X	X
Life satisfaction	Quality of Life Scale	16	X	X
Psychological health	Generalized Anxiety (GAD-7)	7	X	X
	PTSD Checklist (PCL-5)	20	X	X
	Patient Health Questionnaire-9 (PHQ-9)	9	X	X
Unit cohesion	Walter Reed Army Institute for Research (WRAIR) Military Vertical and Horizontal Cohesion Scale	15	X	X
Family functioning	General Functioning Scale (GF12)	12	X	X
Evaluation and feedback about training	Qualitative Interview			2-weeks after training

Assessments will be conducted at baseline and every 4 months thereafter for a total of 16 months.

#### 2.6.1 Measures of psychological resilience

##### 2.6.1.1 Connor-Davidson resilience scale

The CD-RISC (34, 35) is a 25-item questionnaire examining attitudes toward coping with adversity. The shorter, 10-item questionnaire is being administered for this study. The CD-RISC is the primary outcome measure for this study.

##### 2.6.1.2 Dispositional recovery and dysfunction inventory

The DRDI (36) is a 14-item measure comprised of two subscales (recovery and dysfunctional cognitions). Participants rate themselves concerning their perception of themselves on a Likert scale of 1 (not at all characteristic of me) to 7 (entirely characteristic of me).

##### 2.6.1.3 Response to stressful experiences scale

The RSES (37) is a 22-item questionnaire that asks participants to assess how well each statement describes them, both during and after stressful events in their lives.

#### 2.6.2 Measure of trait resilience

##### 2.6.2.1 Short grit scale (Grit-S)

The Grit-S (38) is an eight-item grit scale that examines trait-level perseverance and passion for long-term goals. Participants are asked to rate themselves on items such as “My interests change from year to year” or “I am a hard worker” on a Likert scale ranging from “not at all like me” to “very much like me.”

#### 2.6.3 Measures of psychological flexibility

##### 2.6.3.1 Acceptance and action questionnaire-II

The AAQ-II (39) is a seven-item measure of experiential avoidance and psychological inflexibility. Participants are asked to rate themselves on items such as “Emotions cause problems in my life” on a Likert scale of 1 (never true) to 7 (always true).

##### 2.6.3.2 Multidimensional psychological flexibility inventory

The MPFI (40) is a 60-item measure of the six core processes underlying the ACT psychological flexibility model. Each core process is rated in terms of both flexibility and inflexibility. For each of the six core processes, there are six items reflecting flexibility (acceptance, present moment awareness, self-as-context, cognitive defusion, values, and committed action) and six items reflecting inflexibility (experiential avoidance, lack of contact with the present moment, self-as-content, cognitive fusion, lack of contact with values, and inaction).

#### 2.6.4 Measure of self-efficacy

##### 2.6.4.1 General self-efficacy questionnaire–6

The GSEQ-6 (41) is a six-item, self-report measure that assesses general self-efficacy as related to an individual’s ability to adapt to stressful events.

#### 2.6.5 Measure of mindset

##### 2.6.5.1 Implicit theories of intelligence scale

The Dweck ITIS (42, 43) is a six-item, self-report scale examining individual fixed/growth mindsets concerning intelligence. Participants are asked about their agreement on a 6-point Likert scale from 1 (strongly agree) to 6 (strongly disagree) to items such as, “You have a certain amount of intelligence, and you really cannot do much to change it.”

#### 2.6.6 Measure of life satisfaction

##### 2.6.6.1 Quality of life scale

The QOLS (44) is a 16-item, self-report measure of life satisfaction during the past year in 16 life domains, including health, participation in community and relationships, and creative expression. Items are rated on a scale from 1 (delighted) to 7 (terrible).

#### 2.6.7 Measures of psychological health

##### 2.6.7.1 Generalized anxiety disorder-7 scale

The GAD-7 (45) is a seven-item measure that asks participants to rate the frequency with which they have been bothered by anxiety symptoms within the past 2 weeks on a scale ranging from 0 (not at all) to 3 (nearly every day).

##### 2.6.7.2 PTSD checklist for DSM-5

The PCL-5 (46) is a 20-item, self-report measure of PTSD symptoms, with higher scores reflecting greater PTSD severity. Scoring is based on how much the patient has been bothered by the symptoms in the past month on a scale from 0 (not at all) to 4 (extremely).

##### 2.6.7.3 Patient health questionnaire-9

The PHQ-9 is a widely used and well-validated measure of depressive symptoms (47, 48). It consists of nine items that correspond to the DSM diagnostic criteria for major depressive disorder. Respondents rate the frequency with which they have been bothered by depressive symptoms within the past 2 weeks. Respondents also indicate the degree to which their depressive symptoms have made it difficult for them to do their work, take care of things at home, or get along with other people.

#### 2.6.8 Measure of unit cohesion

##### 2.6.8.1 Walter reed army institute of research (WRAIR) horizontal and vertical cohesion

The WRAIR cohesion scales (49, 50) are the established method of evaluating attitudes about support from peers (horizontal) and leaders (vertical). Horizontal cohesion is assessed using three items, and vertical cohesion is assessed using 13 items. Participant agreement to these 16 statements is rated on a 5-point scale (1 = “strongly disagree” to 5 = “strongly agree”). Individuals answer the questionnaire for their current unit.

#### 2.6.9 Measure of family functioning

##### 2.6.9.1 General functioning 12-item subscale (GF12) of the McMaster family assessment device (FAD)

The GF12 is a 12-item, self-report measure that is a subscale of the FAD (51, 52) designed to assess family functioning. The GF12 subscale includes six items assessing healthy family functioning and six items assessing unhealthy family functioning. Participants are asked about their agreement on a 4-point Likert scale from “strongly agree” to “strongly disagree” to items such as “Planning family activities is difficult because we misunderstand each other” and “In times of crisis we can turn to each other for support.”

#### 2.6.10 Evaluation and feedback about training

##### 2.6.10.1 Qualitative interview

Focus groups will be semi-structured interviews conducted approximately 2 weeks after the training. These groups are designed to assess participants’ perceptions of the training and to solicit recommendations for future training modifications. Qualitative data collected during the focus groups will be recorded and analyzed using a method like that described in the 2015 New Hampshire Medicaid Management Focus Groups (53). Notes and recordings will be transcribed and analyzed for common themes.

### 2.7 Data analytic plan

The primary outcome to address study aims is the change score difference between group (psychological flexibility vs. treatment as usual) on the Connor-Davidson Resilience Scale (CD-RISC) over time. Analyses will be intent-to-treat using data from all randomized participants regardless of the extent of participation. The statistical analysis model is a mixed-effects regression with repeated measures using all assessment time points. Advantages of likelihood-based regression models over conventional ANOVA include the ability to use data from all participants even if they only have baseline data, relaxation of the assumption of equal variances, specification of data distributions other than normal, and the ability to analyze longitudinal data in the presence of missing data. Models will include the fixed effects of group, time, and the respective two-way interaction. Deployment history will also be entered into models as a covariate. Random intercepts and slopes will be tested, and covariance structure selection will be based on likelihood criteria model comparisons (e.g., Akaike’s Information Criteria). Little’s (1988) missing completely at random test and regression-based sensitivity analyses will be used to investigate the nature of missingness and the appropriateness of likelihood-based modeling (54). We will also derive and report on minimally important difference metrics on the CD-RISC (55, 56). Supplementary analyses of the other measures will use the same statistical analysis design. We will report on minimally important change and reliable change indices for the Posttraumatic Stress Disorder Checklist-5 (PCL-5), for which these metrics have been established (55, 57). We will assess deployment history at all time-points and will use this as a covariate in all pre-post analyses. All tests will be two-tailed with unadjusted *p* = 0.05. Analyses will be done using the LME4 package using R statistical software.

#### 2.7.1 Power analysis

Statistical power estimates were obtained from the PASS15 software (NCSS, 2017) modules for comparison of two means or two proportions in a cluster randomized design. Estimates were obtained for a range of standardized mean differences (Cohen’s *d*) from 0.35 to 0.50, which range from modest (*d* = 0.35) to medium in size (*d* = 0.50). Effect sizes below 0.35 are unlikely to be clinically significant and *d* = 0.50 is often considered to be a threshold for a meaningful difference. We also estimated the reduction in Failure % that was detectable at 0.80 assuming a base rate of 80%. We specified a total of 12 clusters of 50 participants for a total *N* = 600. The proposed sample size (*N* = 600) provides statistical power of at least 80% to detect a standardized mean difference (or change over time) in the “small to moderate” range (Cohen’s *d* of 0.30). This applies not only to analyses involving the entire randomized sample but also for analyses based on subsamples based on factors such as deployment, demographics, or other military service characteristics.

#### 2.7.2 Addressing hypotheses

The first research question relates to the pre-post training effect of workshop participation. Analyses will be based on changes during the 4 months before and following the training and will analyze data from all randomized participants. The second research question relates to the effect of training on change during deployments. Analyses will use data only from deployed participants. As deployments occur at varied points in time and are variable in duration, the selection of the baseline and post-deployment assessments for these analyses will be based on the deployment dates of each participant.

#### 2.7.3 Qualitative data analysis

Preliminary themes will be discussed among the study team. Common themes will be developed into a coding scheme. The study team will independently code the interviews using the identified coding scheme. Any differences in coding will be resolved by examining the transcripts. Participants will be invited to participate in a focus group with other participants who engaged in the same type of training (i.e., psychological flexibility training or training as usual). Focus groups will continue until data saturation has occurred and the study team is no longer identifying new themes. Data will be summarized into themes and analyzed using standard qualitative techniques.

### 2.8 Intervention

In coordination with unit leadership, two consecutive days of psychological flexibility training will be integrated into the military training calendar to deliver the intervention to study participants randomized to the intervention arm. The training as usual condition is the U.S. Army’s Master Resiliency Training, which is part of the Comprehensive Soldier Fitness Program (30). Master Resiliency Training is a team training program grounded in principles of positive psychology and strengths-based leadership. Identified soldiers in each unit volunteer to complete master resiliency training and then serve as consultants to units conducting their training as usual resiliency training initiatives.

#### 2.8.1 Psychological flexibility training

Psychological Flexibility Training will be delivered as a 2-day workshop lasting approximately 8 h per day (see Table 2). Table 1 provides an overview of the topics to be covered. An additional description of these concepts as applied to resilience enhancement in the military has been published elsewhere (32). Consistent with the ACT model, experiential exercises will be integrated throughout the training. The primary goals of Day 1 are to provide an overview of the training and to describe the “posture” that prepares one to respond to challenging situations in a psychologically flexible manner. We refer to this as psychological situational awareness. These skills include (1) adopting a position of mindful awareness, (2) increasing clarity regarding one’s core values across different life domains, and (3) adopting an attitude that balances traditional military characteristics such as toughness with acceptance and willingness. This last skill helps to maximize an individual’s ability to cope across the broadest possible range of challenges and life domains and, importantly, to avoid problems associated with engaging an overly narrow or rigid set of coping skills. We also highlight differences between the psychological flexibility stance and some common or “traditional” notions of resilience within military culture. The primary goals of Day 2 are to deepen the rationale for cultivating greater psychological flexibility and to practice core skills based on the psychological flexibility model. Experiential exercises are used to highlight the long-term unworkability of control-based coping attempts (i.e., those based on avoidance and suppression). Psychological flexibility is defined in terms of combining the three elements of (1) contextual sensitivity, (2) awareness of both short and long-term consequences of a given response, and (3) developing and flexibly utilizing an arsenal of skills to meet varied situations and forms of emotional distress. Finally, a series of core psychological flexibility skills are practiced, including noticing and detaching from thoughts, engaging in willingness to experience discomfort, noticing the connection between personal values and the degree of willingness to experience discomfort, and acceptance of emotional distress.

**Table 2:** Overview of psychological flexibility training workshop.

Day 1: Psychological situational awareness		
Module title	Goals	Experiential exercises and metaphors
1. Introduction and overview	Introductions Informed consent for engaging in workshop Discuss the concept of resilience Introduce the resilience “formula” from a psychological flexibility perspective Describe evidence behind the psychological flexibility model	“Two mountains” metaphor to illustrate trainers’ role/stance
2. Position	Define a “position” of resilience to include both posture (attention/intention) and perspective (sense of self) Practice strategies for fully contacting the present moment Increase flexible mindset Expand narrow sense of self to enhance behavioral flexibility	Breathing meditation and practice Mindfulness practices including awareness and attention exercises “Continuous You” meditation for contacting observer self
3. Lunch and mindfulness practice	Practice mindfulness	Mindful eating practice
4. Target	Define personal core values, including how this relates to and is differentiated from Army core values Differentiate values and goals Engage in values-aligned goal setting Differentiate automatically reacting to situations from intentional, values-aligned responding	Values clarification via values card sort exercise Values “target” exercise to assess success in engaging in values-aligned actions across life domains Written values and goals exercise
5. Attitude	Reduce rigidity and influence of cultural programming in the personal definition of psychological strength Increase awareness of unworkable applications of control, particularly when applied toward internal experiences Develop context sensitivity for situations that call for control, change, or acceptance-based coping	“Do not think about…” exercise to demonstrate the limits of cognitive avoidance Discussion of military culture and attitude toward control versus acceptance End of the day small groups debriefing Assignment of awareness-building homework
Day 2: Psychological flexibility		
1. After action review	Review concepts from Day 1 Process homework (noticing emotional distress exercise)	Your ideal 70^th^ birthday – highlights the broad impact of values alignment on life satisfaction
2. Rigidity as the problem	Differentiate between workable versus unworkable control Examine our relationships with our minds Understand the impact of “having” vs. “buying” a thought across situations Identify thoughts that function as rigid rules that may be maladaptive to follow in some contexts	“Anxiety detection machine” – illustrates unworkability of emotion suppression Written exercise to identify control-based coping “Naming your mind” – noticing qualities of the mind “Boxes on a conveyor belt” – noticing and defusing from thoughts “I cannot lift my arm” – highlights thoughts as rules or orders
3. Agility as the alternative	Practice willingness as an alternative to control-based coping Identify short- and long-term outcomes associated with control and acceptance-based coping Develop awareness of the relationship between personal values and emotional pain	“Tug-O-War” exercise to demonstrate the unworkability of control and freedom through emotional willingness Quicksand metaphor to illustrate the context in which acceptance outperforms control/struggle Review the ACT Matrix to establish a framework for guiding values-based action amid aversive internal/external experiences
4. Lunch break	not applicable	not applicable
5. Overcoming psychological obstacles	Practice skills for overcoming unwanted internal experiences Clarify the rationale for developing and engaging a broader range of skills for engaging in values-aligned action in the presence of emotional distress Match skills to different situations and different aspects of distress	“Take your mind for a walk” exercise aimed at noticing and defusing from thoughts “Eyes on” exercise - contacts mindful awareness, self-as-context, defusion, willingness to experience discomfort Acceptance of emotional distress exercise – willingness to experience emotional distress
6. Do what works; Do what matters	Summarize training content Reinforce key principles and practices Provide additional resources including apps, books, and training materials Finalize personal values wallet card Question and Answer	“Passengers on the bus” metaphor to summarize and integrate psychological flexibility processes to facilitate ongoing practice Complete *Resilience Roadmap* worksheet Review and discuss list of psychological flexibility skills with descriptions

To promote retention and practice, participants are provided with handouts summarizing the workshop content, a wallet card that lists their core values identified during the workshop and key points from the workshop, and resources for further reading. In addition, following the workshop, four optional, 1-h booster sessions are offered via video conferencing. The booster sessions are intended to serve as training refreshers and practice/consultation sessions without introducing new content or skills. These sessions follow a standard structure: an opening experiential exercise to highlight one or more processes covered during the workshop, a brief recap of the content presented during the workshop (first booster session only), questions from attendees about applying the concepts in their lives, and responses to questions and additional comments from the facilitators to encourage the application of the concepts.

## 3 Conclusion

Across the DoD, there is an increasing need to train service members to meet worldwide military operational needs. Through collaboration between military leaders and civilian experts in psychological resiliency and applied military research, we believe that the project described will have potential applicability to all U.S. military branches. By combining our collective expertise, we will use the existing scientific evidence to test a culturally competent program designed to enhance resiliency and optimize readiness among active duty service members. For example, Helmreich et al. (14) examined 43 resilience enhancement RCTs and identified numerous “best practices” to implement when developing a resilience enhancement intervention, many of which are implemented herein. Specifically, they posit that a resilience enhancement intervention must describe the underlying resilience concept the intervention is based on (i.e., psychological flexibility within the ACT framework (16)), utilize an *a priori* sample calculation to ensure adequate power, have specified inclusion and exclusion criteria, adequate follow-up periods (herein we utilize four), and conduct a comprehensive baseline assessment. Additionally, the present study uses a detailed randomization process to reduce bias, and a comprehensive assessment battery to maximize the identification of malleable, multi-level resilience factors.

Through the recruitment of many service members (*N* = 600), we will be able to determine the efficacy of the R2OE training program and the extent to which it enhances resiliency and optimizes readiness in comparison to training as usual. The psychological flexibility training, if demonstrated to be effective, will have the potential for dissemination and implementation in other U.S. military units and training communities. In consideration of the potential for widespread implementation, our qualitative interviews will address factors such as motivation to participate in the training, the trainee experience during the workshop, logistical factors that could promote or impede implementation, and both personal and professional impacts of the psychological flexibility training. We view this study design as reflecting a starting point in examining this intervention approach. As such, we chose to use self-report measures of resilience and other outcomes as opposed to biological or behavioral markers, which may be viewed as a limitation of the current study.

Beyond the potential for enhanced military readiness, ACT is broadly applicable and has been demonstrated to be an efficacious intervention across a spectrum of concerns. Psychological flexibility, the central concept within this model, has been described as a fundamental aspect of health (58) any may be synonymous with resilience processes (16, 31). This literature also highlights that ACT is an acceptable intervention that is associated with high trainee satisfaction. We anticipate the present training program could have immediate applicability and benefits for people entering other high-risk occupations. For example, emergency services and medical personnel working in high-stress settings would likely benefit from enhanced psychological flexibility. Psychological flexibility training holds the potential to be a readily modifiable platform to preempt stressor exposure and provide individuals with the psychological tools needed to function effectively in the face of high levels of adversity and emotional distress.

## Ethics statement

The studies involving humans were approved by University of Texas Health Science Center, San Antonio and Brooke Army Medical Center. The studies were conducted in accordance with the local legislation and institutional requirements. The participants provided their written informed consent to participate in this study.

## Author contributions

AP: Conceptualization, Funding acquisition, Investigation, Methodology, Project administration, Resources, Supervision, Writing – review & editing. BM: Investigation, Methodology, Resources, Writing – original draft. WE: Conceptualization, Funding acquisition, Investigation, Writing – review & editing. SY-M: Conceptualization, Funding acquisition, Investigation, Methodology, Project administration, Supervision, Writing – review & editing. AB: Data curation, Formal analysis, Investigation, Supervision, Writing – review & editing. CS: Writing – review & editing. CM: Investigation, Resources, Writing – review & editing. TM: Investigation, Writing – review & editing, Resources. EM: Writing – review & editing, Conceptualization, Funding acquisition, Investigation, Methodology, Project administration, Supervision.

## Glossary

AAQ-II: Acceptance and Action Questionnaire-II
ACT: Acceptance and Commitment Therapy
BCT: Brigade Combat Team
CD-RISC: Connor-Davidson Resilience Scale
DoD: Department of Defense
DRDI: Dispositional Recovery and Dysfunction Inventory
DSM: Diagnostic and Statistical Manual of Mental Disorders
FAD: McMaster Family Assessment Device
GAD-7: Generalized Anxiety Disorder-7 scale
GF12: General Functioning 12-item subscale
Grit-S: Short Grit Scale
GSEQ-6: General Self-Efficacy Questionnaire–6
ITIS: Implicit Theories of Intelligence Scale
IRB: Institutional Review Board
aMPFI: Multidimensional Psychological Flexibility Inventory
PCL-5: PTSD Checklist for DSM-5
PHQ-9: Patient Health Questionnaire
PTSD: posttraumatic stress disorder
QOLS: Quality of Life Scale
R2OE: Resilience and Readiness Optimization/Enhancement
RCT: randomized controlled trial
RSES: Response to Stressful Experiences Scale
SFAB: Security Force Assistance Brigade
UTHSCSA: University of Texas Health Science Center at San Antonio
U.S.: United States
WRAIR: Walter Reed Army Institute of Research cohesion scales
